# Do in-service training materials for midwifery care providers in sub-Saharan Africa meet international competency standards? A scoping review 2000–2020

**DOI:** 10.1186/s12909-022-03772-2

**Published:** 2022-10-14

**Authors:** Joanne Welsh, Hashim Hounkpatin, Mechthild M. Gross, Claudia Hanson, Ann-Beth Moller

**Affiliations:** 1 Midwifery Research and Education Unit, Hannover Medical School, Hannover, Germany; 2 Centre de Recherche en Reproduction Humaine et en Démographie (CERRHUD), Cotonou, Benin; 3 Global Public Health, Karolinska Institute, Stockholm, Sweden; 4 Department of Disease Control, London School of Hygiene and Tropical Medicine, London, UK; 5 School of Public Health and Community Medicine, Institute of Medicine, University of Gothenburg, Gothenburg, Sweden

**Keywords:** Midwifery, Competence, In-service training, Education, Sub-Saharan Africa

## Abstract

**Background:**

Levels of maternal and neonatal mortality remain high in sub-Saharan Africa, with an estimated 66% of global maternal deaths occurring in this region. Many deaths are linked to poor quality of care, which in turn has been linked to gaps in pre-service training programmes for midwifery care providers. In-service training packages have been developed and implemented across sub-Saharan Africa in an attempt to overcome the shortfalls in pre-service training. This scoping review has aimed to summarize in-service training materials used in sub-Saharan Africa for midwifery care providers between 2000 and 2020 and mapped their content to the International Confederation of Midwives (ICM) Essential Competencies for Midwifery Practice.

**Methods:**

Searches were conducted for the years 2000–2020 in Cumulative Index of Nursing and Allied Health Literature, PubMed/MEDLINE, Social Science Citation Index, African Index Medicus and Google Scholar. A manual search of reference lists from identified studies and a search of grey literature from international organizations was also performed. Identified in-service training materials that were accessible freely on-line were mapped to the ICM Essential Competencies for midwifery practice.

**Results:**

The database searches identified 1884 articles after removing duplicates. After applying exclusion criteria, 87 articles were identified for data extraction. During data extraction, a further 66 articles were excluded, leaving 21 articles to be included in the review. From these 21 articles, six different training materials were identified. The grey literature yielded 35 training materials, bringing the total number of in-service training materials that were reviewed to 41. Identified in-service training materials mainly focused on emergency obstetric care in a limited number of sub-Saharan Africa countries. Results also indicate that a significant number of in-service training materials are not readily and/or freely accessible. However, the content of in-service training materials largely met the ICM Essential Competencies, with gaps noted in the aspect of woman-centred care and shared decision making.

**Conclusion:**

To reduce maternal and newborn morbidity and mortality midwifery care providers should have access to evidence-based in-service training materials that include antenatal care and routine intrapartum care, and places women at the centre of their care as shared decision makers.

**Supplementary Information:**

The online version contains supplementary material available at 10.1186/s12909-022-03772-2.

## Background

Despite the target set out by the Sustainable Development Goals (SDG) “to reduce the global maternal mortality ratio (MMR) to less than 70 per 100,000 livebirths by 2030” (SDG 3.1) [1], the global MMR in 2017 was estimated to be 211 per 100,000 livebirths. Of these deaths, 66% were estimated to take place in sub-Saharan Africa, where the estimated regional MMR was 542 in 2017 [2]. Similarly, with a neonatal mortality rate of 27 per 1,000 [3], the sub-Saharan Africa region lags behind the SDG target to reduce neonatal mortality rate to at least as low as 12 per 1,000 livebirths (SDG 3.2) [4]. With access to evidence-based, quality care the majority of these deaths are preventable [5]. Midwifery care, within an enabling environment has the ability to improve quality of care [6, 7]. However, despite a rise in the numbers of women giving birth with skilled health personnel [8], reductions in mortality rates have not fallen proportionately [9]. This may be explained by system deficiencies that influence the provision of high-quality maternity care including a lack of appropriately trained and qualified midwifery care providers.

Pre-service training courses that prepare midwifery care providers for the workforce differ substantially in their length, content and quality [10, 11]. This impacts on the ability of midwifery care providers to perform obstetric and neonatal services [12, 13] and reduces their competence and confidence levels [14]. A form of continuous professional development, in-service training acts to provide updates as new evidence for practice develops, as well as overcome the shortfalls of pre-service training and thereby has the potential to improve quality of care. In sub-Saharan Africa, a number of in-service training packages have been developed and implemented to strengthen the quality of maternity care services [15–17] These have predominantly focused on emergency obstetric care [15–18] with a much smaller number focusing on physiological childbirth [19] and respectful care [20]. In-service training on routine antenatal, intrapartum and postnatal care is basic and vital for positive health outcomes. Midwifery care providers who are trained are competent to identify potential complications and treat them before they develop into more serious and potentially life-threatening complications. To be effective, in-service training materials need to be evidence-based, incorporate up to date local guidelines, and promote autonomy, focusing on multidisciplinary team approaches and woman-centred care. Time needs to be made for midwifery care providers to undertake training to ensure that they can provide evidence based quality of care. The modus in which in-service training is delivered should also be considered as this may influence the impact the training has on care provision in both the short and long term [21].

The International Confederation of Midwives (ICM) “Essential Competencies for Midwifery Practice” outline the minimum set of knowledge, skills and professional behaviour expected of an individual completing their midwifery training when joining the workforce. The ICM Essential Competencies Framework is organised into four inter-related categories; (1) general competencies, (2) pre-pregnancy and antenatal, (3) care during labour and birth and (4) ongoing care for women and newborns. Within each of these, knowledge and skills/behaviour are defined [22]. This Framework is a useful tool in the evaluation and design of previously used, current and future in-service training materials for midwifery care providers.

This scoping review was conducted as part of the Action Leveraging Evidence to Reduce perinatal morTality and morbidity in sub-Saharan Africa (ALERT) project (Trial registration number: PACTR202006793783148) [23]. The objective of the ALERT project is to develop and evaluate a multifaceted intervention to (i) strengthen the implementation of evidence-based interventions and responsive care and, (ii) reduce in-facility perinatal mortality and morbidity through a multidisciplinary approach in Benin, Malawi, Tanzania and Uganda. To achieve this, the ALERT project aims to develop and implement a co-designed in-service midwifery training package with a focus on routine intrapartum care. To inform this process this review has identified in-service training materials for midwifery care providers that will be considered when developing the in-service training packages.

In this review we have used the term “midwifery care provider” which is defined in box 1.

**Box 1:**
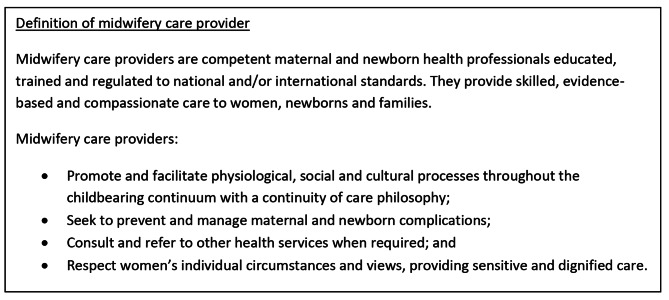
Definition of midwifery care provider (based on Renfrew et al. 2014, WHO 2018 [24, 25])

## Methods

### Study design

The study protocol was published in the BMJ Open in 2021 [26] and outlines the methodology for the design and conduct of the scoping review. Arksey and O’Malley’s [27] scoping review framework was applied in line with the five stages (i) identifying the research question; (ii) identifying relevant studies; (iii) selecting studies; (iv) charting the data; and (v) collating summarizing and reporting the results. This scoping review was conducted to identify in-service training materials used for midwifery care providers in sub-Saharan Africa between 2000 and 2020. It was conducted according to the Preferred Reporting Items for Systematic reviews and Meta-Analyses extension for Scoping Reviews (PRISMA-ScR) Checklist (http://www.prisma-statement.org/Extensions/ScopingReviews), which is outlined in Additional file 1.

#### Stage 1: identifying the research question

All authors discussed and agreed on the research questions. The aim of the scoping review was to summarize existing in-service training materials used in sub-Saharan Africa between 2000 and 2020, and map their content to the ICM Essential Competencies for Midwifery Practice [22] (ICM 2019). Our review posed the following questions:

1. What in-service training materials are available, freely accessible and have been used for midwifery care providers in sub-Saharan Africa from 2000 to 2020?
2. How does the content of these in-service training materials align with the ICM Essential Competencies for Midwifery Practice?

The review sought to include only in-service training materials that are freely available to midwifery care providers. We acknowledge that there are other sponsored and freely available training materials for midwifery care providers. Access to these however, is inequitable as they are only free to those midwifery care providers working in health care facilities where such sponsored training is implemented.

The study protocol outlined that in-service training materials would also be mapped to the Quality Maternal and Newborn Care framework [24]. However, it was noted that the five components of the framework were too general and following further consideration, the decision was made to map the training materials to the ICM Essential Competencies only.

### Stage 2: identifying relevant studies

#### Inclusion criteria

The research questions were assessed, and studies selected specific to the following Population, Concept, Study Design and Context criteria presented in Table 1. All studies and grey literature that included information on available in-service training materials used for midwifery care providers in sub-Saharan Africa were included for review. As the focus of the scoping review was on training materials based on evidence, only those developed and used after 2000 onwards were included as evidence of clinical care changes constantly and new guidelines and recommendations are updated to ensure best practice.

**Table 1:** Inclusion criteria for identifying eligible studies

	Inclusion Criteria
Population	Any in-service training on midwifery care aimed at any health professional who provides midwifery care.
Concept	Mapping the content of in-service training materials used for providers of midwifery care to the ICM Essential Competencies.
Study Design	Studies and reports/documents that report on the implementation of in-service training materials and in-service training resources identified through searches of grey literature.
Context	In-service training in sub-Saharan Africa.

#### Exclusion criteria

Articles were excluded from the scoping review if:

1. The country in which the in-service training took place was not located within sub-Saharan Africa.
2. The health personnel who had undertaken the in-service training were not considered to be midwifery care providers.
3. The content of the in-service training did not relate to the provision of midwifery care.
4. The in-service education and training materials were created before 2000.
5. There were no details provided on the content of the training sessions.
6. The content of the in-service training materials was not freely available online.

#### Search strategy

The search strategy was conducted for all relevant existing literature, without language restrictions, based on search terms relating to the research questions and restricted to the years 2000–2020 using the following online bibliographic databases: Cumulative Index of Nursing and Allied Health Literature, PubMed/MEDLINE, Social Science Citation Index, African Index Medicus, and Google Scholar. Additional file 2 contains terms used for searches of the electronic databases. The search in Google Scholar generated 6,350 results. The authors reviewed the first 150 titles returned by Google Scholar as relevance diminished after this stage.

Grey literature searches were performed and included organizations known to be active in global maternal and newborn health improvement (i.e., United Nations Population Fund (UNFPA), World Health Organization (WHO), Johns Hopkins Program for International Education in Gynecology and Obstetrics (JHPIEGO), International Confederation of Midwives (ICM), International Federation of Gynecology and Obstetrics (FIGO), International Pediatric Association (IPA). The reference list of all eligible studies was hand-searched to identify any additional relevant studies.

#### Stage 3: study selection

Following the searches outlined above, the citations were imported into Covidence (https://www.covidence.org), an online tool developed to support the screening and data extraction processes. The imported citations underwent independent title and abstract screening by two reviewers (A-BM and JW). Screened abstracts identified for inclusion had their full texts independently reviewed by two reviewers (A-BM and JW). Two identified full-texts were written in French and were screened by A-BM. Reasons for exclusion of full texts were documented. See additional file 3 which shows the PRISMA flow diagram charting this process.

#### Stage 4 and 5: charting the data and collating, summarizing and reporting the results

A pre-designed data extraction tool in Microsoft Excel was used to extract data from included studies. Information retrieved included study characteristics (country, year of study and setting). Data collected pertaining to in-service training and educational area of focus, those trained, as well as details related to the design of the in-service training (formal education sessions, training facilitators, on-site training, off-site training, use of simulation, ongoing mentorship, online training), as well as details of identifiable in-service training materials.

JW and HH mapped the training materials to the ICM Competencies, and a third reviewer A-BM randomly checked 20% of the mapped training materials to ensure agreement. To map the content of the in-service training materials to the ICM Essential Competencies for Midwifery Practice, the educational area of focus of the in-service training materials was identified, and content mapped to the corresponding pertinent categories and indicators within the ICM Competencies [22]. Presenting our results using descriptive statistics, as outlined in our protocol paper, was therefore not appropriate, as comparisons between tools would be meaningless. Results are therefore presented using narrative description based on themes arising.

## Results

**Literature search**.

The search of electronic databases yielded 2009 articles. Removal of 125 duplicates left 1884 articles whose titles and abstracts were screened independently by two reviewers. A total of 170 articles were selected for full text review. The full text review led to a further 83 articles being excluded. The most common reason for exclusion was “not in-service training” meaning the study did not report on in-service training. See the PRISMA diagram in additional file 3 for full details of the reasons for exclusions. A further 66 articles were excluded during the data extraction phase as the in-service training materials were not identifiable and/or were not freely available. Corresponding authors were contacted in cases where the materials could not be located. Of those that responded the following responses were given; authors no longer had access to the training materials and advised us to contact the organization who had developed the training materials. This organization was contacted but did not respond; some authors could not share the training materials as they are not freely available to the public; and one author was able to share a scanned copy of the first few pages of the training materials. Following a search, we were able to obtain a soft copy of the training materials from a colleague working in the low-and-middle-income country where the materials had been developed. However, as these materials were not freely available online, we did not include them in the review.

Websites of organizations known to be active in maternal and newborn global health improvement (i.e., UNFPA, WHO, JHPIEGO, ICM, FIGO, IPA) were searched. This process identified a further 44 in-service training materials. However, during the mapping phase, nine of these were excluded. In one third of these cases, the reason for exclusion was difficulty in accessing the training materials online. Where possible, the appropriate organizations were contacted and asked for support to obtain, but no responses were received.

### Study characteristics

Details of the studies [28–48] included in the review are provided in Table 2. Included studies were concentrated in eight countries, Ethiopia, Ghana, Kenya, Malawi, Mali, Sudan, Tanzania and Uganda with one third of the studies reporting on in-service training in Tanzania. Two studies reported on the implementation of in-service training across multiple countries (including India, Kenya, Malawi and Tanzania).

**Table 2:** Included articles

Author(s)	Title	Publication Year	Country	Participants	Setting	Topic	Identified Training Material
Yigzaw et al. [28]	Comparing the effectiveness of a blended learning approach with a conventional learning approach for basic emergency obstetric and newborn care training in Ethiopia.	2019	Ethiopia	Midwives, nurses, health officers	Health centres.	BEmONC	Federal Democratic Republic of Ethiopia Ministry of Health: Basic Emergency Obstetric and Newborn Care
Weinberg et al. [29]	Helping Babies Survive Training Programs: Evaluating a Teaching Cascade in Ethiopia.	2019	Ethiopia	Midwives, nurses, house officers	Urban and rural. Hospitals.	Neonatal Resuscitation and Neonatal Care	American Academy of Pediatrics: Helping Babies Breathe, Essential Care for Every Baby, Essential Care for Small Babies
Thomsen et al. [30]	Health workers’ experiences with the Safe Delivery App in West Wollega Zone, Ethiopia: a qualitative study.	2019	Ethiopia	Nurses, midwives, health extension workers	Urban and rural. Health centres and hospitals.	BEmONC, EmONC, Neonatal Resuscitation	Safe Delivery App
Lund et al. [31]	Association Between the Safe Delivery App and Quality of Care and Perinatal Survival in Ethiopia: A Randomized Clinical Trial.	2016	Ethiopia	Nurse, midwives, health extension workers	Urban and rural. Health centres and hospitals.	BEmONC, EmONC, Neonatal Resuscitation	Safe Delivery App
Mirkuzie et al. [32]	Standard basic emergency obstetric and neonatal care training in Addis Ababa; trainees’ reaction and knowledge acquisition.	2014	Ethiopia	Nurses and midwives	Urban. Health centres.	BEmONC	Federal Democratic Republic of Ethiopia Ministry of Health: Basic Emergency Obstetric and Newborn Care.
Eblovi et al. [33]	Retention and use of newborn resuscitation skills following a series of helping babies breathe trainings for midwives in rural Ghana.	2017	Ghana	Midwives	Rural. Health centres.	Neonatal Resuscitation	American Academy of Pediatrics: Helping Babies Breathe
Innerdal et al. [34]	Reduction in Perinatal Mortality after Implementation of HBB Training at a District Hospital in Mali.	2020	Mali	Birth attendants	Rural. Hospitals and health centres.	Neonatal Resuscitation	American Academy of Pediatrics: Helping Babies Breathe
Draiko et al. [35]	Knowledge, skills and competency retention among health workers one year after completing helping babies breathe training in South Sudan.	2019	Sudan	Medical officers/doctors, nurses, midwives, maternal child health officers, community health workers, and clinical officers	Urban. Hospitals.	Neonatal Resuscitation	American Academy of Pediatrics: Helping Babies Breathe
Arabi et al. [36]	Perinatal outcomes following Helping Babies Breathe training and regular peer-peer skills practice among village midwives in Sudan.	2018	Sudan	(Village) Midwives	Rural. Medical centres.	Neonatal Resuscitation	American Academy of Pediatrics: Helping Babies Breathe
Arabi et al. [37]	Skills retention in Sudanese village midwives 1 year following Helping Babies Breathe training.	2016	Sudan	(Village) Midwives	Rural. Medical centres.	Neonatal Resuscitation	American Academy of Pediatrics: Helping Babies Breathe
Alwy Al-Beity et al. [38]	Predictors of change of health workers’ knowledge and skills after the Helping Mothers Survive Bleeding after Birth (HMS BAB) in-facility training in Tanzania.	2020	Tanzania	Doctors, nurse-midwives, auxiliary providers	Rural. Hospitals and health centres.	Bleeding after Birth	JHPIEGO (ICM, FIGO, UNFPA, WHO, MCSP, AAP): Helping Mothers Survive Bleeding After Birth
Egenberg et al. [39]	Impact of multi-professional, scenario-based training on postpartum hemorrhage in Tanzania: a quasi-experimental, pre- vs. post-intervention study.	2017	Tanzania	Nurse-midwives, doctors	Rural. Hospitals.	Bleeding after Birth	JHPIEGO (ICM, FIGO, UNFPA, WHO, MCSP, AAP): Helping Mothers Survive Bleeding After Birth
Egenberg et al. [40]	“No patient should die of PPH just for the lack of training!“ Experiences from multi-professional simulation training on postpartum hemorrhage in northern Tanzania: a qualitative study.	2017	Tanzania	Nurse-midwives, doctors, and medical attendants	Urban. Hospitals.	Bleeding after Birth	JHPIEGO (ICM, FIGO, UNFPA, WHO, MCSP, AAP): Helping Mothers Survive Bleeding After Birth
Neilssen et al. [41]	Clinical performance and patient outcome after simulation-based training in prevention and management of postpartum haemorrhage: an educational intervention study in a low-resource setting.	2017	Tanzania	Nurse-midwives, medical attendants (nurse aides without formal medical education), and ambulance drivers (without formal education)	Rural. Hospitals.	Bleeding after Birth	JHPIEGO (ICM, FIGO, UNFPA, WHO, MCSP, AAP): Helping Mothers Survive Bleeding After Birth
Wilson et al. [42]	Helping Babies Breathe implementation in Zanzibar, Tanzania.	2017	Tanzania (Zanzibar)	Nurse-midwives, clinical officers	Urban and rural. Clinics.	Neonatal Resuscitation	American Academy of Pediatrics: Helping Babies Breathe
Neilssen et al. [43]	Helping mothers survive bleeding after birth: an evaluation of simulation-based training in a low-resource setting.	2014	Tanzania	Nurse-midwives, medical attendants (nurse aides without formal medical education), and ambulance drivers (without formal education)	Rural. Hospitals.	Bleeding after Birth	JHPIEGO (ICM, FIGO, UNFPA, WHO, MCSP, AAP): Helping Mothers Survive Bleeding After Birth
Mesmo et al. [44]	Newborn mortality and fresh stillbirth rates in Tanzania after helping babies breathe training.	2013	Tanzania	Health care providers	Urban. Hospitals.	Neonatal Resuscitation	American Academy of Pediatrics: Helping Babies Breathe
Williams et al. [45]	“Practice so that the skill does not disappear”: mixed methods evaluation of simulator-based learning for midwives in Uganda.	2019	Uganda	Doctors, nurse, midwives and “others”	Urban and rural. Hospitals and health centres.	Neonatal Resuscitation and Bleeding after Birth	American Academy of Pediatrics: Helping Babies Breathe. JHPIEGO (ICM, FIGO, UNFPA, WHO, MCSP, AAP): Helping Mothers Survive Bleeding After Birth
Hanson et al. [46]	Effects of the Helping Mothers Survive Bleeding after Birth training on near miss morbidity and mortality in Uganda: A cluster-randomized trial.	2021	Uganda	Maternity providers	Rural. Hospitals and health centres.	Bleeding after Birth	JHPIEGO (ICM, FIGO, UNFPA, WHO, MCSP, AAP): Helping Mothers Survive Bleeding After Birth
Bang et al. [47]	Helping Babies Breathe (HBB) training: What happens to knowledge and skills over time?	2016	Multi-county (India and Kenya)	Nurses and doctors (India). Nurse-midwives (Kenya)	Hospitals.	Neonatal Resuscitation and Neonatal Care	American Academy of Pediatrics: Helping Babies Breathe
Evans et al. [48]	Competency-based training “Helping Mothers Survive: Bleeding after Birth” for providers from central and remote facilities in three countries.	2014	Multi-country (India, Malawi, Tanzania incl. Zanzibar)	Health orderlies (Zanzibar only), Auxiliary nurse midwife (India only), Nurses/nurse- midwives (all 3 countries), Clinical officers, medical assistants, and doctors (all 3 countries)	Urban and rural. Health facilities.	Bleeding after Birth	JHPIEGO (ICM, FIGO, UNFPA, WHO, MCSP, AAP): Helping Mothers Survive Bleeding After Birth

### In-service training materials

From the 21 studies included in the review, six in-service training materials were identified. Two studies reported on the implementation of more than one in-service training material. The most commonly implemented in-service training materials used were the American Academy of Pediatrics “Helping Babies Breathe” (n = 10) and the JHPIEGO “Helping Mothers Survive – Bleeding After Birth” (n = 8). In countries where more than one study took place it was noted that the same training materials were repeatedly implemented. In Sudan for example, there were three studies, all of which reported on the use of the American Academy of Pediatrics “Helping Babies Breathe”. Similarly, five of the seven studies in Tanzania reported on JHPIEGO’s “Helping Mothers Survive Bleeding After Birth”.

Table 3 shows all 41 in-service training materials identified from the literature search and the grey literature search of websites of organizations known to be active in maternal and newborn global health improvement. The authors grouped the in-service training materials into eight main categories; family planning, antenatal care, intrapartum care, postnatal care - women, postnatal care – newborn, combined in-service training materials (materials that covered aspects of care across the antenatal, intrapartum and postnatal continuum), post abortion care, and whole life course (generic topics relevant to all aspects of care). There was a lack of in-service training materials focused solely on antenatal care (n = 4) and intrapartum care (n = 8). Sub-categories were used to identify training materials focused on uncomplicated and complicated aspects of antenatal care, intrapartum care, and postnatal care for women and the newborn. In these categories there was an equal or higher number of materials focusing on complications compared to uncomplicated antenatal, intrapartum and postnatal care. Additionally, all in-service training materials in the combined in-service training materials category, contained some elements related to the provision of care for complications.

**Table 3:** Identified in-service training materials

Educational area of focus	ID No.	Training package title	Author	Year	Type of material	Link to material
Family planning	1	Immediate contraception post obstetrical event (ICPOE) – 2021	Pan American Health Organization	2021	Online learning resource	Immediate contraception post obstetrical event (ICPOE) − 2021 | Virtual Campus for Public Health (VCPH/PAHO) (https://www.campusvirtualsp.org)
	2	Postpartum family planning	Global Health Learning Centre	2016	Online learning resource	Postpartum Family Planning | Global Health eLearning Center (https://www.globalhealthlearning.org)
	3	Updates on contraceptive technology Part 1	Geneva Foundation for Medical Education and Research	2013	Online learning resource	https://www.gfmer.ch/SRH-Course-2013/family-planning/pdf/Updates-contraceptive-technology-Part1-Vogelsong-Festin-2013.pdf
	4	Updates on contraceptive technology Part 2	Geneva Foundation for Medical Education and Research	2013	Online learning resource	Updates on contraceptive technology. Part 2 (https://www.gfmer.ch)
	5	Workshop on Comprehensive Postpartum Family Planning Care	JHPIEGO (USAID and ACCESS Family Planning Initiative)	2008	Soft copy - available online	http://www.reprolineplus.org/resources/workshop-comprehensive-postpartum-family-planning-care-learning-resource-package
Antenatal care - Uncomplicated	6	Antenatal care	Global Health Learning Centre	2005	Online learning resource	Antenatal Care | Global Health eLearning Center (https://www.globalhealthlearning.org)
	7	Basic Maternal and Newborn Care: Basic Antenatal Care	JHPIEGO/Maternal and Neonatal Health Program	2004	Soft copy - available online	Antenatal Care Module: Course Handbook for Participants: Basic Maternal and Newborn Care Learning Resource Package | ReproLinePlus / Antenatal Care Module: Course Notebook for Trainers: Basic Maternal and Newborn Care Learning Resource Package | ReproLinePlus (https://reprolineplus.org/)
Antenatal care - Complicated	8	Prevention and Control of Malaria in Pregnancy (3rd edition)	JHPIEGO	2018	Soft copy - available online	Prevention and Control of Malaria in Pregnancy, Third Edition, 2018 Update | ReproLinePlus(https://reprolineplus.org/)
	9	Malaria in pregnancy	Global Health Learning Centre	2012	Online learning resource	Malaria in Pregnancy | Global Health eLearning Center (https://www.globalhealthlearning.org)
Labour care -Uncomplicated	10	Helping Mothers Survive: Essential Care for Labour and Birth	JHPIEGO	2019	Soft copy - available online. Also available as online learning resource	Helping Mothers Survive Essential Care for Labor & Birth (African Graphics) | ReproLinePlus(https://reprolineplus.org/)
	11	Alternative Birth Positions	Maternal and Child Survival Program	2016	Soft copy -available online	Alternative Birth Positions training materials (https://www.mcsprogram.org)
Labour care - Complicated	12	Helping Mothers Survive: Pre-eclampsia and Eclampsia	JHPIEGO	2017	Soft copy - available online. Also available as online learning resource	Helping Mothers Survive Pre-Eclampsia & Eclampsia Training Package: English (African Graphics) | ReproLinePlus (https://reprolineplus.org/)
	13	Helping Mothers and Babies Survive: Threatened Preterm birth care	JHPIEGO	2016	Soft copy - available online	http://reprolineplus.org/HMS-PTB-LRP
	14	Managing Prolonged and Obstructed Labour	JHPIEGO	2012	Online learning resource	Jhpiego: Log in to the site (https://learning.jhpiego.org/login/index.php)
Postnatal - Mother - Uncomplicated	15	Postpartum care	Global Health Learning Centre	2019	Online learning resource	Postpartum Care – Maternal Health Task Force (https://www.mhtf.org)
Postnatal - Mother - Complicated	16	Helping Mothers Survive: Bleeding After Birth	JHPIEGO	2017	Soft copy - available online. Also available as online learning resource	Helping Mothers Survive Bleeding After Birth Complete Training Package: English (International Graphics) | ReproLinePlus / (https://reprolineplus.org/)www.learning.jhpiego.org
	17	The evidence-based management of postpartum haemorrhage	Geneva Foundation for Medical Education and Research	2013	Online learning resource	The evidence-based management of Postpartum Haemorrhage (https://www.gfmer.ch)
	18	Managing Postpartum Haemorrhage	JHPIEGO	2012	Online learning resource	Jhpiego: Log in to the site (https://learning.jhpiego.org/login/index.php)
	19	Managing Puerperal Sepsis	JHPIEGO	2012	Online learning resource	Jhpiego: Log in to the site (https://learning.jhpiego.org/login/index.php)
Postnatal - Baby - Uncomplicated	20	Helping Babies Survive: Essential Care for Every Baby	American Academy of Pediatrics (not freely available from AAP, but freely available from UNHCR)	2014	Soft copy - available online	UNHCR - Essential Care For Every Baby - Provider Guide (ENG) (https://www.unhcr.org/publications/brochures/5db073c64/essential-care-baby-provider-guide-eng.html?query=essential%20care%20of%20every%20baby)
	21	Essential Newborn Care	JHPIEGO	2012	Online learning resource	Jhpiego: Log in to the site (https://learning.jhpiego.org/login/index.php)
	22	Cord Care	Ghana Health Service and USAID, Maternal and Child Survival Program (on JHPIEGO website)	Unknown	Online learning resource	Jhpiego: Log in to the site (https://learning.jhpiego.org/login/index.php)
Postnatal - Baby - Complicated	23	Helping Babies Survive: Essential Care for Small Babies	American Academy of Pediatrics	2015	Soft copy - available online	UNHCR - Helping babies survive - Essential care for small babies: Facilitator Flip Chart (ENG) (https://www.unhcr.org/publications/brochures/5e14a8724/helping-babies-survive-essential-care-small-babies-facilitator-flip-chart.html?query=helping%20babies%20survive)
	24	Care of low-birth-weight babies through Kangaroo Mother Care	JPHIEGO	2015	Soft copy - available online	http://reprolineplus.org/resources/KMC-LRP
	25	Helping Babies Breathe	American Academy of Pediatrics	2016	Soft copy - available online	UNHCR - Helping Babies Breathe - Facilitator Flip Chart (ENG) (https://www.unhcr.org/publications/brochures/5db040554/helping-babies-breathe-facilitator-flip-chart-eng.html?query=helping%20babies%20survive)
	26	Managing Newborn Problems: A guide for midwives, doctors and nurses.	JHPIEGO	2004	Soft copy - available online	http://reprolineplus.org/resources/managing-newborn-problems-guide-midwives-doctors-and-nurses-learning-resource-package
Combined (antenatal, intrapartum, postnatal)	27	Safe Delivery App	Maternity Foundation. University of Copenhagen. University of Southern Denmark.	Constantly updated	App available online	Need to download
	28	Basic Emergency Obstetric and Newborn Care	Federal Democratic Republic of Ethiopia. Ministry of Health	2013	Soft Copy - available online	Final BEmONC -Training package.pdf (https://www.ethernet.edu.et)
	29	Best Practices in Maternal and Newborn Care: A learning resource package for essential and basic emergency obstetric newborn care	JHPIEGO	2008	Soft copy - available online	Best Practices in Maternal and Newborn Care: A Learning Resource Package for Essential and Basic Emergency Obstetric and Newborn Care | ReproLinePlus (https://reprolineplus.org/)
	30	Life-Saving Skills Manual for Midwives	American College of Nurse-Midwives	2008	Soft Copy - available online	ACNM Global Publications | ACNM Publications (https://www.midwife.org)
	31	Basic Maternal and Newborn Care: A guide for skilled providers	JHPIEGO	2004	Soft Copy - available online	Basic Maternal and Newborn Care: A Guide for Skilled Providers (https://www.reprolineplus.org) http://reprolineplus.org/system/files/resources/bp_mnc_ppts_0.pdf
Post Abortion Care	32	Post abortion care	JHPIEGO	2012	Online learning resource	Jhpiego: Log in to the site (https://learning.jhpiego.org/login/index.php)
	33	Post abortion care	JHPIEGO (USAID, ACCESS)	2010	Soft copy - available online	Post abortion Care Learning Resource Package | ReproLinePlus (https://reprolineplus.org/)
Whole Life course	34	ICM Respect Workshop	International Confederation of Midwives	2020	Soft copy -available online	Respect Toolkit (https://www.internationalmidwives.org)
	35	Infection Prevention and Control Module 1: Introduction to Infection Prevention and Control	JHPIEGO	2018	Soft copy - available online	Infection Prevention and Control. Module 1: Introduction to Infection Prevention and Control | ReproLinePlus (https://reprolineplus.org/)
	36	Infection Prevention and Control Module 2: Hand Hygiene	JHPIEGO	2018	Soft copy - available online	Infection Prevention and Control. Module 2: Hand Hygiene | ReproLinePlus (https://reprolineplus.org/)
	37	Maternal infections	Geneva Foundation for Medical Education and Research	2015	Online learning resource	Maternal Infections e-Learning Course (https://www.gfmer.ch)
	38	Gender Based Violence	JHPIEGO	Unknown	Online learning resource	Jhpiego: Log in to the site (https://learning.jhpiego.org/login/index.php)
	39	Maternal and child vaccinations	JHPIEGO	Unknown	Online learning resource	Jhpiego: Log in to the site (https://learning.jhpiego.org/login/index.php)
	40	Maternal nutrition	JHPIEGO	Unknown	Online learning resource	Jhpiego: Log in to the site (https://learning.jhpiego.org/login/index.php)
	41	Nurses and midwives’ contribution to an HIV free generation	JHPIEGO	Unknown	Online learning resource	Jhpiego: Log in to the site (https://learning.jhpiego.org/login/index.php)

### Approaches used to conduct in-service training

Table 4 outlines approached used to conduct the in-service training in the 21 studies included in the scoping review. Of note, whilst studies reported on how in-service training was conducted, the finer details relating to facilitation of learning were limited. One study reported in-service training taking place off-site, 12 reported on training taking place onsite and eight studies did not specify where the training took place. Face to face lectures were used in 20 of the 21 studies, with only three studies reporting the use of e-learning. Nineteen studies reported that simulation was used as a teaching tool, whilst almost half (10 studies) reported that ongoing mentorship was used to support in-service training.

**Table 4:** Approaches used to conduct in-service training

Author(s)	Country	Identified Training Material	Location of training	Online lectures	Face to face lectures	Simulation	Mentor- ship	E-learning
Yigzaw et al. [28]	Ethiopia	Federal Democratic Republic of Ethiopia Ministry of Health: Basic Emergency Obstetric and Newborn Care.	Offsite		x	x	x	
Weinberg et al. [29]	Ethiopia	American Academy of Pediatrics: Helping Babies Breathe, Essential Care for Every Baby, Essential Care for Small Babies	Onsite		x	x		
Thomsen et al. [30]	Ethiopia	Safe Delivery App	Unknown		x			x
Lund et al.	Ethiopia	Safe Delivery App	Unknown		x			x
Mirkuzie et al. [32]	Ethiopia	Federal Democratic Republic of Ethiopia Ministry of Health: Basic Emergency Obstetric and Newborn Care.	Unknown		x	x		
Eblovi et al. [33]	Ghana	American Academy of Pediatrics: Helping Babies Breathe	Unknown		x	x	x	
Innerdal et al. [34]	Mali	American Academy of Pediatrics: Helping Babies Breathe	Onsite			x		
Draiko et al. [35]	Sudan	American Academy of Pediatrics: Helping Babies Breathe	Unknown		x	x	x	
Arabi et al. [36]	Sudan	American Academy of Pediatrics: Helping Babies Breathe	Onsite		x	x	x	
Arabi et al. [37]	Sudan	American Academy of Pediatrics: Helping Babies Breathe	Onsite		x	x	x	
Alwy Al-Beity et al. [38]	Tanzania	JHPIEGO: Helping Mothers Survive Bleeding After Birth	Onsite		x	x	x	
Egenberg et al. [39]	Tanzania	JHPIEGO: Helping Mothers Survive Bleeding After Birth	Onsite		x	x		x
Egenberg et al. [40]	Tanzania	JHPIEGO: Helping Mothers Survive Bleeding After Birth	Unknown		x	x		
Neilssen et al. [41]	Tanzania	JHPIEGO: Helping Mothers Survive Bleeding After Birth	Unknown		x	x		
Wilson et al. [42]	Tanzania (Zanzibar)	American Academy of Pediatrics: Helping Babies Breathe	Onsite		x	x		
Neilssen et al. [43]	Tanzania	JHPIEGO: Helping Mothers Survive Bleeding After Birth	Unknown		x	x		
Mesmo et al. [44]	Tanzania	American Academy of Pediatrics: Helping Babies Breathe	Onsite		x	x	x	
Williams et al. [45]	Uganda	American Academy of Pediatrics: Helping Babies Breathe. JHPIEGO: Helping Mothers Survive Bleeding After Birth	Onsite		x	x	x	
Hanson et al. [46]	Uganda	JHPIEGO: Helping Mothers Survive Bleeding After Birth	Onsite		x	x	x	
Bang et al. [47]	Multi-county (India and Kenya)	American Academy of Pediatrics: Helping Babies Breathe	Onsite		x	x	x	
Evans et al. [48]	Multi-country (India, Malawi, Tanzania incl. Zanzibar)	JHPIEGO: Helping Mothers Survive Bleeding After Birth	Onsite		x	x		

### Alignment with the ICM essential competencies for midwifery practice

The content of the in-service training materials largely met the ICM Essential Competencies [22]. However, the in-service training materials largely lacked focus on woman-centred care and shared decision making. Additional file 4 contains the mapped in-service training materials.

## Discussion

To our knowledge this is the first scoping review to map the content of in-service training materials to the ICM Essential Competencies for midwifery practice [22]. Whilst we found the content of the training materials to mostly align to the pertinent ICM Competencies, a lack of focus on woman-centred care and shared decision-making was noted. This finding is in line with recent evidence that suggests rather than being woman-centred, maternity care in sub-Saharan Africa is institution centred [49]. Evidence from Malawi and Ghana indicates that midwifery care providers do not explain to women the reasons for procedures or ask for consent prior to carrying out procedures and furthermore, that women do not feel they can ask questions [50, 51]. This finding is not isolated to countries in sub-Saharan Africa. In high-income countries women-centred care has been the rhetoric since the 1990s, but women still report that they are not as involved in decision making about their care as they would like [52]. This finding is important as it highlights an important gap in in-service training content. Evidence suggests uptake of skilled birth attendance is influenced by respectful, woman-centred care. Using in-service training to ensure the current midwifery workforce are both educated about, and able to provide respectful woman-centred care, may help to increase skilled birth attendance, and therefore improve maternal and newborn outcomes [53].

There are 46 countries in sub-Saharan Africa, yet our scoping review found published literature reporting on in-service training for midwifery care providers in only eight of these countries (Ethiopia, Ghana, Kenya, Malawi, Mali, Sudan, Tanzania and Uganda). The lack of reports from other countries could be because in-service training is not provided or that no research on in-service training is performed. Still, it begs the question as to whether input and resources from high income countries are being centralised to the few rather than the many. This scoping review underlines the previous finding that where in-service training is provided, the focus is on emergency obstetric care with little focus on routine intrapartum care. This is justifiable on the grounds that obstetric haemorrhage is still one of the leading causes of maternal mortality in low-income countries [54]. However, this raises the question as to the focus of in-service training. Preventative and promotive midwifery care that has the potential to improve maternal and neonatal outcomes has largely been overlooked. A study conducted in Uganda revealed that midwifery care providers failed to provide appropriate antenatal, intrapartum and postnatal care for the 872 women enrolled in their study [55]. With routine care standards falling below WHO recommendations, strategies need to be found to improve quality of care provision [55]. Our findings therefore provide clear guidance that routine antenatal, intrapartum and postnatal care need greater attention on the in-service training agenda to ensure women and newborns receive needed and timely care to prevent emergency situations. High quality antenatal care for example, has the potential to support women to adopt a healthy lifestyle, prepare for childbirth, and furthermore, allows midwifery care providers to identify potential risks that may compromise the ongoing pregnancy and birth [56]. Equally, education on routine intrapartum care may help midwifery care providers identify complications during labour, which if acted on, may prevent a number of obstetric emergencies from occurring. With the main focus of included studies being emergency obstetric care, and in particular Helping Mothers Survive Bleeding After Birth, it is interesting to note that despite all this training, postpartum haemorrhage remains a leading cause of maternal death in sub-Saharan Africa. This questions the effectiveness of current in-service training programmes. Whilst evidence from Tanzania suggests training and on-site clinical mentorship improves the self-reported performance of midwifery care providers, further research is needed to assess the long-term effects of such training [21].

Where information was provided, it was noted that the majority of in-service training took place onsite with face-to-face lectures and simulation used as the main teaching techniques. Whilst a strong body of evidence reveals that in-service training has a limited impact on improving outcomes [18, 57], there is evidence to suggest that the use of low-dose-high-frequency simulation techniques improve the efficacy of in-service training [58]. Given the limited impact in-service training has on quality of care and maternal and newborn outcomes, it could be argued that resources aimed at improving the knowledge, skills and behaviours of midwifery care providers should be steered towards improving pre-service education and training. However, improvements to pre-service education and training will not be felt by the current midwifery care provider workforce. It is therefore essential that despite its documented limitations, in-service training that focuses on routine antenatal, intrapartum and postnatal care should continue. In providing the current workforce with the opportunity to develop their knowledge and clinical skills, improvements may be seen in the provision of evidence-based, quality care, which in turn has the potential to reduce maternal and newborn morbidity and mortality [18, 57, 58].

Implementing and gathering evidence on the implementation and outcomes of in-service training could be viewed as altruistic. It is surprising therefore, that in excess of 50 studies were excluded from this scoping review because the in-service training materials they reported on were not easily and/or freely available. Of the studies included, the majority provide little detail on how in-service training was conducted. Failing to report on and evaluate methods of educational approach reduces the ability of others to identify effective practice or learn from possible good practice/mistakes as they go on to implement in-service training. Moving forwards and in light of the current COVID-19 pandemic, the use of e-learning methodologies may be required to ensure midwifery care providers can continue to be trained to perform evidence-based care.

Finally, although not a main focus of this scoping review, very little information was provided on the costs of implementing in-service training, thereby limiting the possibility to calculate the cost-effectiveness of such an intervention. Collectively, these issues limit the ability of midwifery care providers to learn from previous experiences of implementing in-service education, thereby reducing the possibility for successful training approaches to be replicated.

### Strengths and limitations of the review

The main strength of this review is that it is the first study to our knowledge that provides an overview of available in-service training materials used in sub-Saharan Africa between 2000 and 2020. Furthermore, it is the first study to our knowledge that maps the content of in-service training materials to the ICM competencies. Limitations of the review include our inability to access and review all in-service training materials used in sub-Saharan Africa between 2000 and 2020. Furthermore, we have been unable to review fully the approaches used to conduct in-service training.

## Conclusion

This scoping review found that the majority of in-service training materials identified focused on emergency care for postpartum haemorrhage, and that studies reporting their use were concentrated in a small number of African countries. To reduce maternal and newborn mortality across sub-Saharan Africa, it is essential that midwifery care providers in all countries have access to evidence-based in-service training. The review revealed that a significant number of in-service training materials are not readily and/or freely available. If we truly want to meet the targets of the SDGs and improve maternal and newborn care and outcomes, in-service training materials that are successful in improving provider knowledge and skills, need to be shared and readily available for use. The content of the identified in-service training materials largely met the ICM Essential Competencies, with gaps noted in the aspect of woman-centred care and shared decision making. Placing women at the centre of their care and involving them in decision making may improve their satisfaction with care and their desire to access care. Incorporating aspects of woman-centred care into future in-service training materials should therefore be a priority. It is also important that in-service training is not used as a substitute for poor pre-service training, and the quality of pre-registration courses should be such that midwifery care providers are competent professionals when they qualify.

In conclusion, to reduce maternal and newborn morbidity and mortality midwifery care providers should have access to evidence-based in-service training that includes antenatal care and routine intrapartum care, and places women at the centre of their care as shared decision makers.

## Electronic supplementary material

Below is the link to the electronic supplementary material.

Supplementary Material 1

Supplementary Material 2

Supplementary Material 3

Supplementary Material 4

## Data Availability

The datasets supporting the conclusions of this article are included within the article (and its additional files).

## List of abbreviations

**FIGO**: International Federation of Gynecology and Obstetrics.
**ICM**: International Confederation of Midwives.
**IPA**: International Pediatric Association.
**JHPIEGO**: Johns Hopkins Program for International Education in Gynecology and Obstetrics.
**MMR**: Maternal mortality ratio.
**UNFPA**: United Nations Population Fund.
**WHO**: World Health Organization.
