# Supplementary material for: Do in-service training materials for midwifery care providers in sub-Saharan Africa meet international competency standards? A scoping review 2000–2020
Source: BMC Med Educ. 2022 Oct 14;22:725. doi: 10.1186/s12909-022-03772-2 (PMC9568981; doi:10.1186/s12909-022-03772-2)
Supplement: Supplementary file 2 — Supplementary Material 2 [file 12909_2022_3772_MOESM2_ESM.docx]

**Additional file 2: Database searches.**

**Do in-service training materials for midwifery care providers in sub-Saharan Africa meet international competency standards? A scoping review 2000-2020.**

**Database searches conducted on 29^th^ April 2021**

African Medicus Index – 5 Results

tw:(tw:((tw:(midwifery)) OR (tw:(maternity)) OR (tw:(obstetrics))) AND tw:((tw:(inservice training)) OR (tw:(simulation )) OR (tw:(low dose high frequency)) OR (tw:(clinical skills)) OR (tw:(competency based training))) AND ( collection_gim:("AIM")) AND (year_cluster:[2000 TO 2021])) AND (year_cluster:[2000 TO 2021])

CINAHL – 433 Results (restricted to 2000-current date)

(((MH "Midwifery+") OR (MH "Students, Nurse Midwifery") OR (MH "Students, Midwifery") OR (MH "Lay Midwifery") OR (MH "Research, Midwifery") OR (MH "Nurse-Midwifery Service") OR (MH "Nurse Midwifery") OR (MH "Midwifery Service+")) AND ((MH "Professional Knowledge+") OR (MH "Clinical Competence+") OR (MH "Professional Competence+") OR (MH "Competency Assessment") OR (MH "Competency Assessment") OR (MH "Education, Competency-Based") OR (MH "Teaching Methods+/EV") OR (MH "Critical Thinking/EV")) AND (impact OR evaluation OR tools OR measuring OR exam* OR test* OR impact*)) OR (MH "Education, Midwifery" AND (evaluation OR impact OR tools OR testing OR exam* OR competen* OR "critical thinking") )

Google Scholar – 6,350 results generated. First 150 titles viewed. Included/exported 18 to Covidence

| With **all** of the words | Midwifery, Africa |
| --- | --- |
| With the **exact phrase** |  |
| With **at least one** of the words | Inservice training, competency-based training, clinical skills, simulation, low dose high frequency |
| **Without** the words |  |
| Where my words occur |  |
| Return articles **authored** by |  |
| Return articles **published** by |  |
| Return articles **dated** between | 2000-2021 |

Social Sciences Index (Web of Science) – 202 Results

| **Search No.** | **Search Term** | **No. of results 28.04.2021 (with limit of 2000-2021)** |
| --- | --- | --- |
| **1** | Midwifery | 38,573 |
| **2** | Maternity | 16,272 |
| **3** | Obstetrics | 417,953 |
| **4** | Inservice training | 191 |
| **5** | Competency based training | 5,836 |
| **6** | Clinical skills | 54,848 |
| **7** | Simulation | 1,703,765 |
| **8** | Low dose high frequency | 9,328 |
| **9** | Low and middle income | 27,809 |
| **10** | Africa | 419,846 |
| **11** | #1 OR #2 OR#3 | 462,901 |
| **12** | #4 OR #5 OR #6 OR #7 OR #8 | 1,767,015 |
| **13** | #9 OR #10 | 441,498 |
| **14** | #11 AND #12 AND #13 AND #14 | **202** |

PubMed – 1351 Results

| **Search No.** | **Search Term** | **29.04.2021(with data limit of 2000-now)** |
| --- | --- | --- |
| **1** | Midwifery (MeSH Major Topic) |  |
| **2** | Maternity |  |
| **3** | Obstetrics (MeSH Major Topic) |  |
| **4** | Inservice training |  |
| **5** | Competency based training |  |
| **6** | Clinical skills |  |
| **7** | Simulation |  |
| **8** | Low dose high frequency |  |
| **9** | Africa | 389,686 |
| **10** | #1 OR #2 OR #3 | 478,850 |
| **11** | #4 OR #5 OR #6 OR #7 OR #8 | 937,243 |
| **12** | #9 AND #10 AND #11 | **1351** |

**Total results imported into Covidence = 2009**
