# Supplementary material for: Do in-service training materials for midwifery care providers in sub-Saharan Africa meet international competency standards? A scoping review 2000–2020
Source: BMC Med Educ. 2022 Oct 14;22:725. doi: 10.1186/s12909-022-03772-2 (PMC9568981; doi:10.1186/s12909-022-03772-2)

Additional file 3: PRISMA Flow Diagram

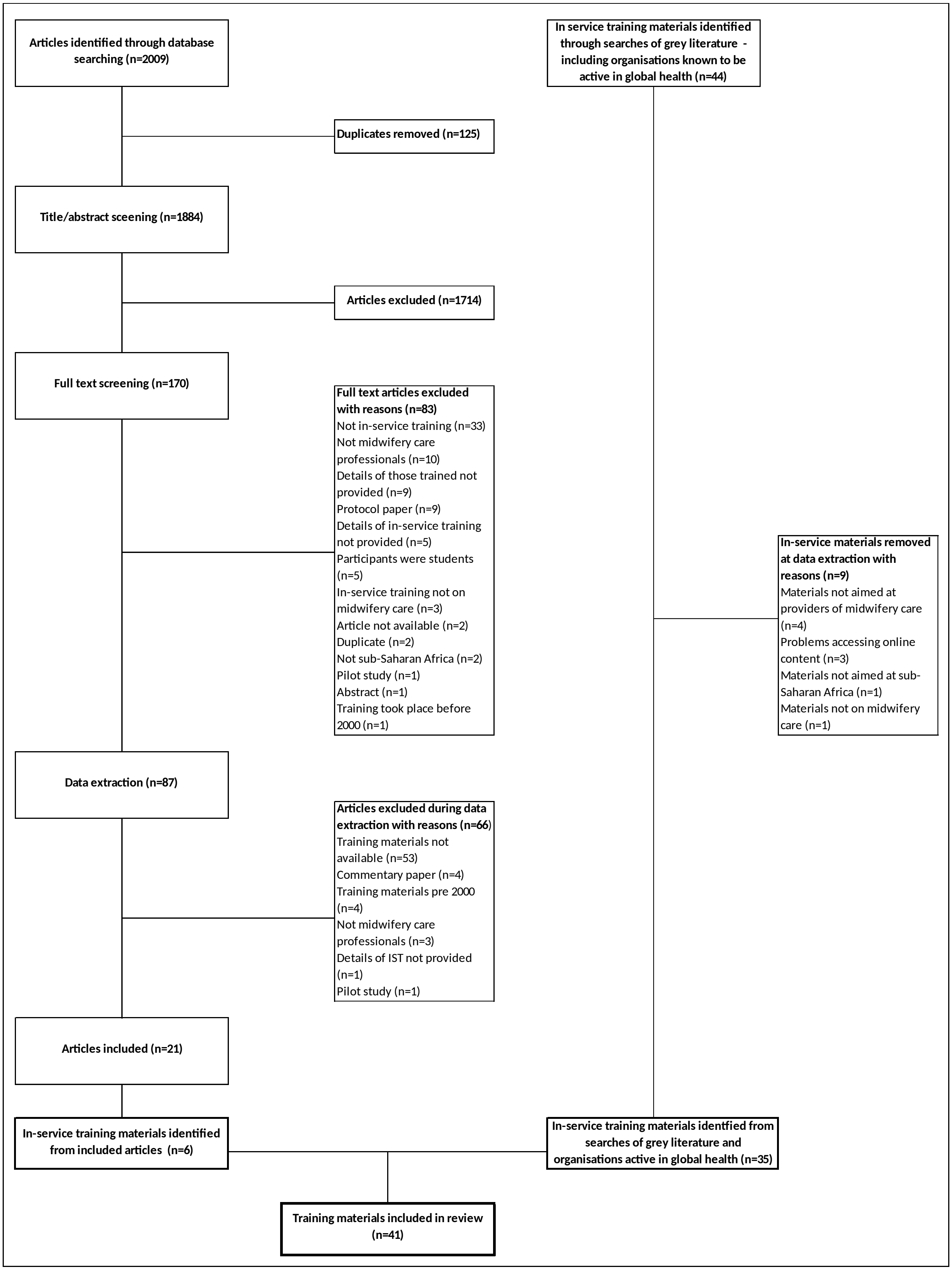

Supplement: Supplementary file 3 [file 12909_2022_3772_MOESM3_ESM.docx]
